# Recruiting the microcirculation in septic shock

**DOI:** 10.1186/s13613-019-0577-9

**Published:** 2019-09-11

**Authors:** Matthieu Legrand, Daniel De Backer, François Dépret, Hafid Ait-Oufella

**Affiliations:** 1 Department of Anesthesiology and Perioperative Care, University of California, San Francisco, USA; 2 AP-HP, GH Saint Louis-Lariboisière, Department of Anesthesiology and Critical Care and Burn Unit, University Paris Diderot, Paris, France; 3 UMR INSERM 942, Institut National de la Santé et de la Recherche Médicale (INSERM), Paris, France; 4 F-CRIN, INICRCT Network, Nancy, France; 5 Department of Intensive Care, CHIREC Hospitals, Université Libre de Bruxelles, Brussels, Belgium; 6 Department of Critical Care, AP-HP, Saint Antoine Hospital, Paris, France; 7 INSERM U970, Paris Cardiovascular Center, Paris, France

Mortality of critically ill patients is driven by severity of the insult, comorbidities, appropriate treatment, and organ failure. The pathophysiological mechanisms leading to organ failure during sepsis remain poorly understood. The direct toxicity of pro-inflammatory mediators and/or bacterial components and/or circulating particles released from damaged cells has been suggested to contribute to organ dysfunction. Alterations of microcirculatory perfusion were associated with organ failure severity and mortality in septic shock patients. Tissue perfusion is likely to play a role in the development of organ dysfunction either due to systemic or regional alterations. Interestingly, organ dysfunction may persist despite apparent restoration of systemic macrohemodynamic variables [1]. Optimal macrohemodynamic targets to perfuse and recruit the microvessels may vary, however, between patients and between vascular beds [2].

Altogether, these associations suggest that intra-organ blood flow defects might be (at least partially) causal in the development of organ failure and targeting the microcirculation might promote organ recovery and better outcome. The question of which therapeutics may be implemented based on peripheral perfusion or microcirculation assessment is central. While animal studies have shown a beneficial impact of treatment targeting the microcirculation on organ blood flow and damage recovery, clinical data are scarcer [3]. Therapeutic intervention can help improving organ perfusion in selected patients. Fluid loading was shown to improve microcirculatory blood flow when applied early on during the course of sepsis [4] or in the operating room [5]. Dobutamine improved microvascular perfusion in some patients but not in others [6]. In post-cardiopulmonary bypass shock, increasing the perfusion pressure (PP) by increasing the mean arterial pressure (MAP) from 60 to 75 mmHg using norepinephrine could increase renal blood flow and glomerular filtration rate. Variability in the best PP for optimizing renal blood flow, however, further underlined the variability in response to treatments. Some patients did improve their renal hemodynamic while other deteriorated while further increasing MAP from 75 to 90 mmHg [7]. Others showed that increasing the MAP above 65 mmHg did not improve microperfusion or even could worsen it in some patients [8–10]. A vasopressor challenge in patients with multiple organ failure remaining hypoperfused despite fluids (i.e. oliguria, increased capillary refilling time, mottling, cold extremities) can work out the best perfusion pressure to recruit microvessels. Monitoring the perfusion response to an increase of PP is, however, warranted to avoid overtreatment in unresponsive patients. Transfusion of red blood cells can increase the microcirculatory flow and the proportion of perfused vessels after an haemorrhagic shock [11] or in some but not all septic patients with anaemia [12]. In any case, a wide variability exists between patients regarding the impact of fluids and vasopressors to recruit the microcirculation. The benefit of improving cardiac output and increasing MAP on the microcirculation appear prominent when these parameters and the capillary perfusion are altered at baseline before the therapeutic challenge. Yet imaging monitoring tools (i.e. side-dark field imaging) have revealed that microcirculatory disorders may persist despite the apparent normalization of macrohemodynamic variables.

Among the mechanisms implicated in the impairment of microcirculatory blood flow (i.e. intravascular thrombosis, endothelial dysfunction, increased extravascular tissue pressure), altered balance between levels of vasoconstrictive and vasodilating substances may be sensitive to vasodilatory agents [13]. An important study from De Backer et al. highlighted that topical administration of acetylcholine, a powerful endothelium-dependent vasodilating agent, restored the sublingual microcirculatory blood flow in septic shock patients suggesting a role for vasodilating agents [14]. In an animal model, administration of l-arginine could restore the microcirculation when combined with vasopressors during endotoxemia [15]. These findings suggest that inappropriate vasoconstriction may be central in the sepsis-associated microperfusion reduction and this could be totally reversed, opening an area for vasodilator-based therapeutic intervention.

Whether intravascular administration of vasodilator agents acting at the resistive arterioles may improve capillary blood flow remains to be determined (Fig. 1) [16]. We propose to illustrate the intense heterogenous vasoconstriction of resistive arterioles, associated with a drop of the critical closing pressure in some territories as the “bottleneck-like vascular barrier” (Fig. 1). This theory may therefore reconcile the con-intuitive approach of providing both vasopressors and vasodilators in distributive shock. Selecting the best vasodilating drug and the best population that could benefit from vasodilating drug are major issues. De Backer et al. showed that dobutamine could recruit the microcirculation in septic patients, independently of the cardiac response, through its vasodilating action [6, 17]. In a randomized study including 70 septic shock patients, nitroglycerin did not provide any benefit at the microcirculation level and tend to worsen prognosis [18]. However, nitroglycerin yields significant venous dilatory properties. In addition the trial mostly included patients with uncompromised sublingual microcirculatory blood flow so that the improvement in microcirculation was expected to be very limited. Prostacyclin analogues are potent arteriolar vasodilators that may fulfil the criteria of predominantly acting at the precapillary level. Experimental data suggest that prostacyclin derivatives may improve microvascular perfusion [19]. Prostacyclin analogue administration has shown promising results in shock patients [20] and is currently investigated in a multicentre randomized controlled trial (NCT03788837).

**Fig. 1:**
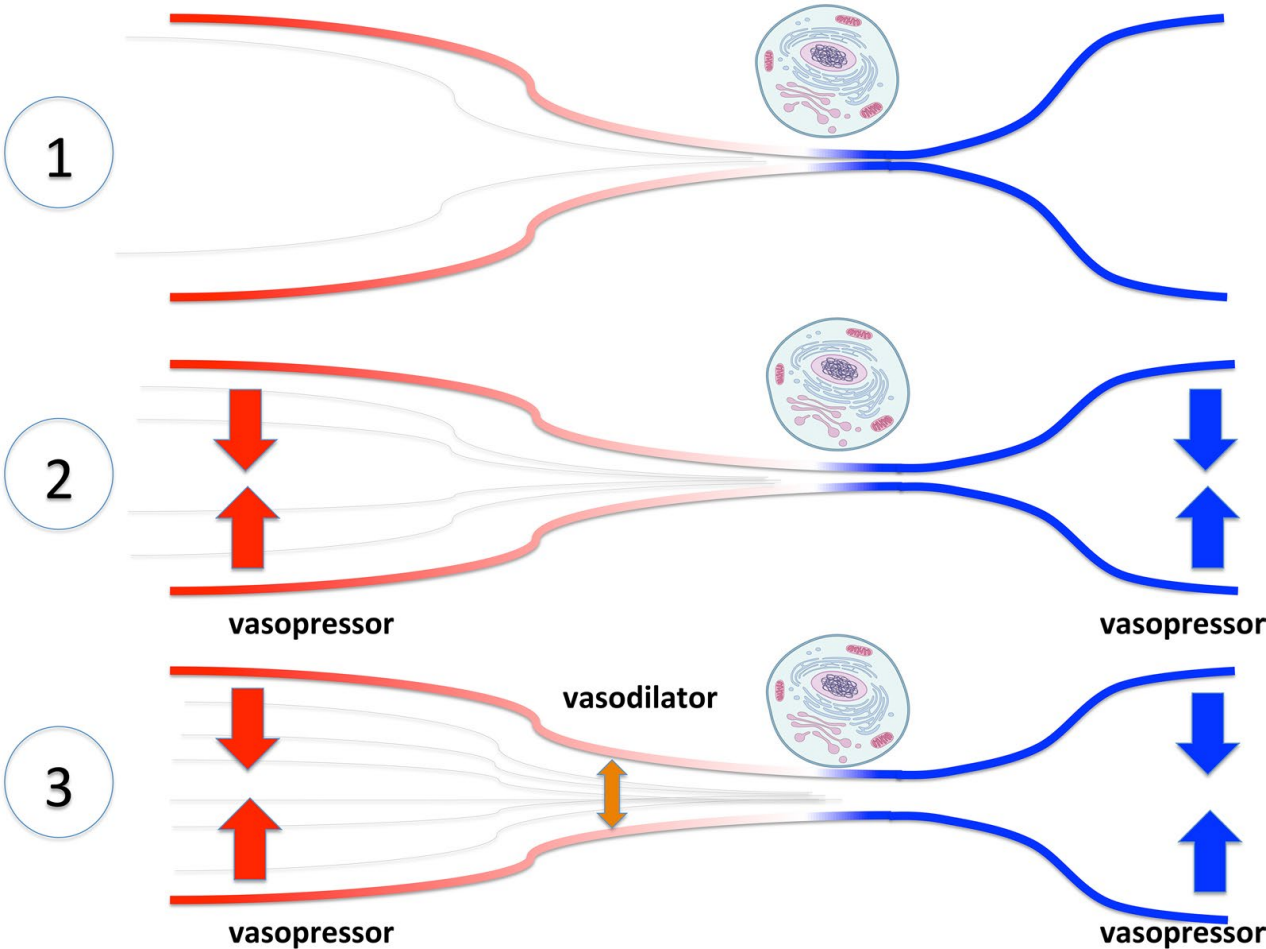
Schematic representation of microvascular recruitment strategies of microvessels in distributive shock. Impaired tissue perfusion in distributive shock may be associated with low arterial pressure due to systemic vasodilation and intense vasoconstriction of precapillaries arterioles and external compression of capillaries in some vascular territories (Panel 1). Vasopressor agents increase inflow pressure by arteriolar vasoconstriction. Cardiac output can also increase due to venous constriction. Perfusion pressure may not increase despite the arterial pressure rise if the increase in perfusion pressure is insufficient to overcome precapillary vascular resistance and its closing critical pressure (Panel 2). This obstruction—theorized as a vascular bottleneck—might be targeted using vasodilators, which may improve microcirculatory flow and oxygen supply in selected patients (Panel 3)

Selecting the right patients for the right treatments in a tailored microcirculatory-targeting strategy will be mandatory. At the bedside, skin mottling around the knee area, prolonged capillary refill time, central-to-peripheral gradient temperature are the easily and frequently observed manifestations of microperfusion disorders [21]. These clinical parameters correlated with organ failure severity and are predictive of ICU mortality and may help guiding the treatment. A recent large-scale trial demonstrated that capillary refill time can be used to guide early resuscitation [22]. However, the resuscitations measures were mostly oriented toward the systemic circulation. Future well-designed investigations should answer the question whether strategies aimed at recruiting the microcirculation may be triggered by these variables and may improve outcome. Yet, these clinical signs might fail to explore the heterogeneity of microperfusion between vascular beds and organs but also inside some organs. Selecting the patients with a profoundly altered microcirculation unresponsive to conventional therapy is crucial. “Easy to use, easy to learn” clinical signs are available and can identify patients with severe peripheral tissue hypoperfusion. Such investigations will answer the question whether we should target the “microcirculation bottleneck” in shock patients.

## Data Availability

Not applicable.
