# Correction to: Recruiting the microcirculation in septic shock

**DOI:** 10.1186/s13613-019-0581-0

**Published:** 2019-09-24

**Authors:** Matthieu Legrand, Daniel De Backer, François Dépret, Hafid Ait-Oufella

**Affiliations:** 1 Department of Anesthesiology and Perioperative Care, University of California, San Francisco, USA; 2 AP‑HP, GH Saint Louis‑Lariboisiere, Department of Anesthesiology and Critical Care and Burn Unit, University Paris Diderot, Paris, France; 3 UMR INSERM 942, Institut National de la Santé et de la Recherche Médicale (INSERM), Paris, France; 4 F-CRIN, INICRCT Network, Nancy, France; 5 Department of Intensive Care, CHIREC Hospitals, Université Libre de Bruxelles, Brussels, Belgium; 6 Department of Critical Care, AP-HP, Saint Antoine Hospital, Paris, France; 7 INSERM U970, Paris Cardiovascular Center, Paris, France

## Correction to: Ann. Intensive Care (2019) 9:102 10.1186/s13613-019-0577-9

After publication of the original article [1], we were notified that an author’s name has been incorrectly spelled. Author’s first name is Hafid and the family name is Ait-Oufella (with a hyphen between Ait and Oufella).

The original article has been corrected.
